# “Smartphone Medication Adherence Saves Kidneys” for Kidney Transplantation Recipients: Protocol for a Randomized Controlled Trial

**DOI:** 10.2196/13351

**Published:** 2019-06-21

**Authors:** John McGillicuddy, Jessica Chandler, Luke Sox, Martina Mueller, Lynne Nemeth, Prabhakar Baliga, Frank Treiber

**Affiliations:** 1 College of Medicine Medical University of South Carolina Charleston, SC United States; 2 College of Nursing Medical University of South Carolina Charleston, SC United States

**Keywords:** mHealth, kidney transplant, medication adherence, digital health

## Abstract

**Background:**

Kidney transplant recipients’ poor medication adherence and poor control of comorbidities, particularly hypertension, are risk factors for graft rejection, graft loss, and death. Few randomized controlled trials (RCTs) have been successful in improving sustained medication adherence and blood pressure control among kidney transplantation recipients. We provide rationale for an RCT evaluating a mobile health medical self-management system for kidney transplantation recipients called Smartphone Medication Adherence Saves Kidneys (SMASK).

**Objective:**

Our objective is to determine whether SMASK is efficacious in improving medication adherence and sustaining blood pressure control among kidney transplantation recipients with uncontrolled hypertension and poor medication adherence compared to an enhanced standard care.

**Methods:**

This two-arm, 6-month, phase II single-site efficacy RCT will involve 80 kidney transplantation recipients. Participants will be randomly assigned to the SMASK intervention arm or control arm. SMASK includes multilevel components: automated reminders from an electronic medication tray; tailored text messages and motivational feedback, guided by the self-determination theory; and automated summary reports for providers. Evaluations will be conducted preintervention, at 3 and 6 months, and posttrial at 12 months. Specific aims are to test the hypotheses that compared to standard care, the SMASK cohort will demonstrate significantly improved changes at 3, 6, and 12 months in the primary outcome variables medication adherence (proportion with electronic monitor-derived score >0.90) and blood pressure control (proportion meeting and sustaining adherence to the Kidney Disease Improving Global Outcomes [KDIGO] guidelines for blood pressure control); the secondary outcome variables provider adherence to KDIGO guidelines, measured by timing of medication changes and changes in self-determination theory constructs; and the exploratory outcome variables estimated glomerular filtration rate, variability in calcineurin inhibitor trough levels, and proportion of patients meeting and sustaining the 24-hour ambulatory blood pressure below 130/80 mm Hg. After the 6-month evaluation, interviews with a random sample of SMASK subjects (n=20) and health care providers (n=3-5) will assess user reactions including acceptability, usability, and aids/barriers to sustainability. Data from the RCT and interviews will be triangulated to further refine and optimize SMASK and prepare for a multisite effectiveness RCT.

**Results:**

The SMASK project received funding from National Institute of Diabetes and Digestive and Kidney Diseases in June 2016, obtained institutional review board approval in April 2016, and began data collection in July 2016. As of July 2018, we completed enrollment with a total of 80 participants.

**Conclusions:**

This study will provide data regarding the efficacy of SMASK to improve medication adherence and blood pressure control in a cohort of hypertensive kidney transplant recipients. An efficacious SMASK intervention will pave the way for a larger, multicenter, effectiveness RCT powered sufficiently to evaluate clinical events in a real-world setting and with the potential to demonstrate improved outcomes at lower cost than standard care.

**International Registered Report Identifier (IRRID):**

DERR1-10.2196/13351

## Introduction

End-stage renal disease (ESRD) affects more than 700,000 people living in the United States; of these, approximately 95,000 are currently awaiting kidney transplantation [1,2]. Kidney transplantation is the treatment of choice for eligible patients with ESRD, as it offers superior quality of life, improved life expectancy, and better psychosocial functioning, all at a lower cost than maintenance hemodialysis [3-6].

Advances in the medical and surgical care of transplant recipients have not resulted in optimal long-term graft survival. The current 5-year graft survival rate is only 78% [2], and the average graft half-life is only ~9 years [7]. Poor medication adherence and poor control of comorbid medical conditions, particularly hypertension, are major drivers of suboptimal kidney transplantation outcomes [8-13]. Nonadherence to prescribed medical regimens has been identified as a primary risk factor for graft rejection, graft loss, and death [14-18]. Even small degrees of nonadherence to immunosuppressant medications confer a significantly increased risk of graft rejection or graft loss [16,18]. In a meta-analysis published in 2007, approximately 35% of American kidney transplantation recipients demonstrated nonadherence to medications posttransplantion [19], with other more recent studies reporting values of 20%-40% [20-22].

Although medication adherence is critical for optimal kidney transplantation outcomes, until relatively recently, there was a paucity of research examining interventions directed at improving adherence. Our formative research has shown that kidney transplantation recipients have a high rate of smart mobile phone ownership, are comfortable being monitored using mobile health (mHealth) technology, and have an overall positive attitude toward mHealth [23,24]. We previously conducted a small, 3-month, two-arm randomized controlled trial with 20 kidney transplant recipients that involved a patient-centered, behavioral theory–guided mHealth intervention (Smartphone Medication Adherence Saves Kidneys [SMASK]). SMASK included tailored motivational/social reinforcement short message service (SMS) messages, an electronic medication tray with cellular connectivity and reminder alerts, and Bluetooth-enabled blood pressure (BP) self-monitoring, designed to improve both medication adherence and BP control [25]. The recruitment process included confirmation of poor medication adherence using an electronic medication tray (<80% over 1-month monitoring) and documented uncontrolled hypertension prior to and following the 1-month screening process. The SMASK group demonstrated significantly greater improvements in electronically calculated medication adherence (average of 0.92 vs 0.56, *P*<.50) and guideline-based systolic BP (SBP) control (90% vs 10%, *P*<.50) across the 3-month trial compared to the standard care (SC) control group. A recent 12-month posthoc follow-up of the subjects’ clinic SBPs demonstrated persistence of the SBP difference between the groups (132 mm Hg vs 154 mm Hg), suggesting that the improvement in medication adherence was sustained in the intervention group [26].

Investigation on this topic has dramatically increased. A 2017 review of medication adherence intervention trials performed in solid organ transplant recipients identified 21 randomized controlled trials (RCTs), with 15 involving kidney transplant recipients. Two of the studies identified were our 3-month RCT [25] and the subsequent 12-month post trial follow-up study [27]. Intervention approaches in kidney transplant recipient studies varied widely and included cognitive behavioral therapy aimed at improving medication adherence [28,29]; psychoeducation [30]; intensified pharmaceutical care [31]; financial assistance programs [32]; electronic monitoring and reminders [33-35]; and our mHealth system that provides reminders, tracks medication taking, and delivers tailored motivational/social reinforcement messages based on the level of adherence.

Although approximately half of these RCTs involving kidney transplant recipients demonstrated a significant improvement in medication adherence, and the methodologies and measures of adherence varied widely. Most either did not examine drug blood levels or could not demonstrate an improvement in the examined levels related to medications(s) taken. None of the five RCTs in kidney transplant recipients that examined transplant outcomes (ie, estimated glomerular filtration rate [eGFR], graft rejection, graft survival, and serum creatinine) demonstrated significant improvement. None of these RCTs lasted >12 months, and the incidence rates of rejection and other relevant parameters were too infrequent to allow comparisons. Importantly, although poorly controlled hypertension is highly prevalent (70%-93%) [8,10,11] and a leading contributor to posttransplant reductions in kidney function, changes in associated physiological function (eg, BP) were not measured in any RCT except ours [26].

The authors concluded that research on medication adherence programs in the transplant population is misguided in that it often does not include patients verified as having poor medication adherence, lacks the use of programs that are engaging and foster sustained regimen adherence, and does not evaluate clinically relevant transplant-specific outcomes. Our work has addressed several of these issues including identification and recruitment of kidney transplant recipients with verified poor medication adherence using multiple indices. We chose to employ a two-part screening process including BP measurement and one-time medication adherence via medication possession ratio screening followed by a 1-month screening to determine medication nonadherence via a Bluetooth-enabled electronic pill box with audio and visual alerts disabled. In a pilot study, we found that 70% of otherwise eligible kidney transplant recipients were medication nonadherent [25], and thus far, we have identified a higher (~82%) proportion of such recipients in this RCT. Although medication nonadherence is on a continuum, it has been well demonstrated that even small degrees of medication nonadherence have negative effects on kidney transplant recipient outcomes [16-18]. We have employed a patient-centered, theory-guided, iterative design process to develop a medical regimen self-management program aimed at fostering self-efficacy and autonomous motivation to help ensure regimen adherence is sustained. In light of the expense and length of follow-up necessary to demonstrate significant clinical outcome improvements (eg, reduced acute rejection, graft loss, and death), we have adopted a strategy to investigate surrogate markers that are shown to be strongly predictive of worsened long-term outcomes (ie, BP and percent coefficient of variation [%cv]) [36-44]. A recent review of the literature on hypertension management in kidney transplant recipients concluded that posttransplant hypertension is prevalent (70%-90%), multifactorial, and rarely controlled (~33%) [8]. Specifically, among kidney transplant recipients at our transplant center, we found the prevalence of hypertension to be 95%, with only 34% having met the Kidney Disease Improving Global Outcomes (KDIGO) guidelines for hypertension control at a mean follow-up of 7.3 (SD 4.5) years after transplant [45]. Uncontrolled hypertension remains a significant problem in kidney transplant recipients; thus, efficacious intervention programs are warranted.

This paper describes a 6-month efficacy RCT with a 6-month posttrial follow-up, utilizing a further refined SMASK mHealth system in kidney transplant recipients verified to be medication nonadherent with poorly controlled hypertension. The aims of this study are twofold: (1) to assess the efficacy of our mHealth system for monitoring and enhancing medication adherence and BP control during the 6-month active trial and the 6-month post-trial follow-up period and (2) to evaluate provider adherence to BP management guidelines and changes in participants’ levels of self-efficacy and autonomous motivation to sustain engagement in the medical regimen over time. Exploratory outcome variables will include changes in eGFR and variability of calcineurin inhibitor (CNI) trough levels (%cv).

## Methods

### Trial Design

**Figure 1:**
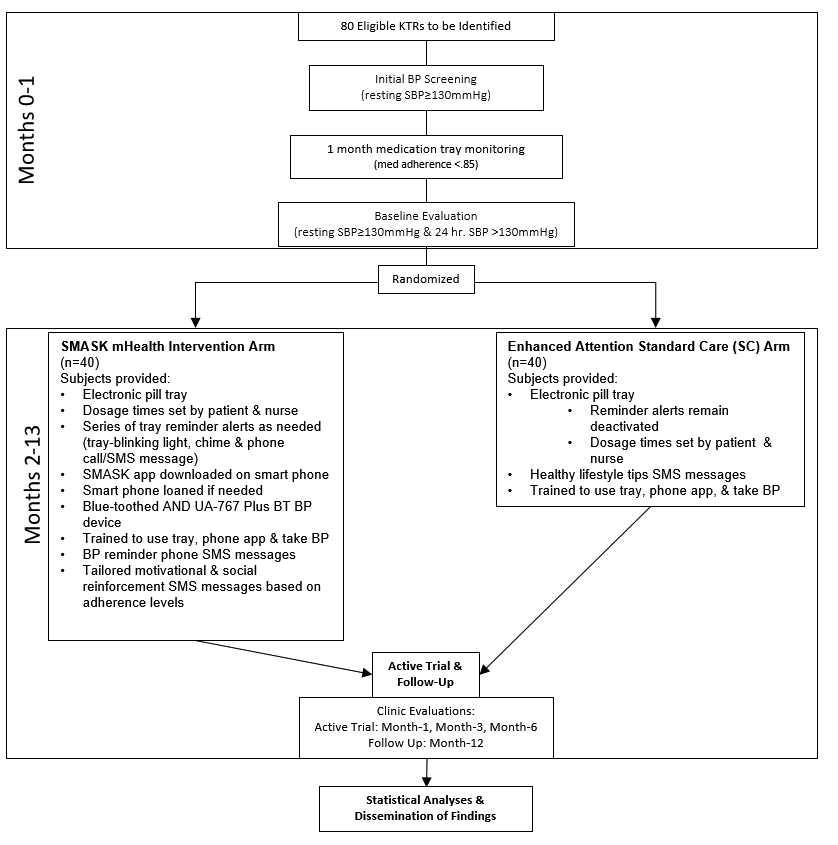
SMASK clinical trial flowchart. SMASK: Smartphone Medication Adherence Saves Kidneys; SBP: systolic blood pressure; BP: blood pressure; SMS: short message service; KTR: kidney transplant recipient.

This is a two-arm, phase II efficacy RCT involving 80 kidney transplant recipients with poor medication adherence and uncontrolled hypertension, with subjects as the unit of randomization (ClinicalTrials.gov: NCT02827695). Each of the subjects was recruited and randomly assigned to the intervention or control arm by a statistician (who is not involved in subject recruitment or data collection) using a computer-generated random sequence. A flow chart of the study design is presented in Figure 1.

### Study Setting

This study is conducted at the Medical University of South Carolina (MUSC) in the Kidney Transplant Clinic. MUSC is a tertiary referral academic center in Charleston, SC, and the only transplant center in the state.

### Study Participants

The participants are adult kidney transplant recipients with uncontrolled hypertension who have met the inclusion/exclusion criteria. Potentially eligible study patients were identified through weekly data extractions from the appointment database.

The inclusion criteria for participants were age >18 years (male or female), first- or second-time recipient of a functioning solitary kidney transplantation performed 6 weeks to 60 months earlier, legally competent, assent provided by the transplant physician so that the patient is able to participate, patient prescribed ≥3 medications for immunosuppression and hypertension, electronic pill tray–based medication adherence score <0.85 across a 1-month screening period, uncontrolled systolic hypertension (SBP ≥130 mm Hg) at the screening visit and baseline evaluation (following 1-month medication monitoring period), and 24-hour ambulatory SBP ≥130 mm Hg following the medication monitoring period.

The exclusion criteria were severe cognitive impairment/dementia; inability to self-administer medications, self-measure BP, or use a mobile phone; poor cellular coverage at home; inability to speak, hear, or understand English; history of psychiatric illness or substance abuse; planned pregnancy; and vulnerable populations such as pregnant or nursing women, prisoners, and institutionalized individuals.

### Prototype of the Mobile Health Smartphone Medication Adherence Saves Kidneys System

**Figure 2:**
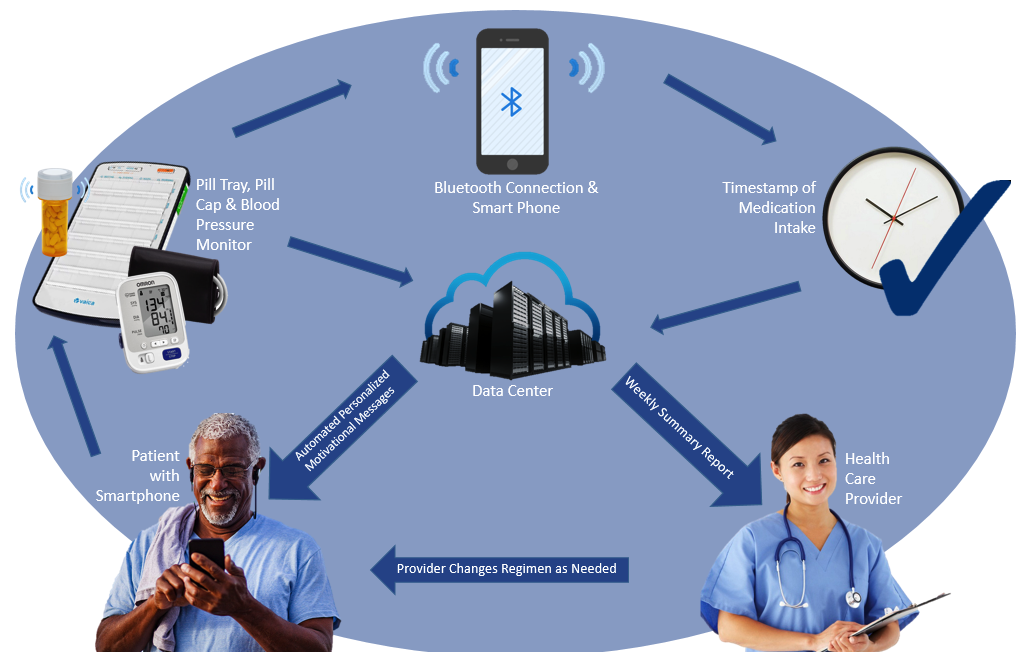
“Smartphone Medication Adherence Saves Kidneys” schematic.

The SMASK mHealth system characterized in Figure 2 consists of a wireless global system for mobile communication electronic medication tray (Vaica, Tel Aviv, Israel), a wireless validated Bluetooth low energy–enabled BP monitor (UA-767 Plus BT device [46]), and a smartphone (Android running Lollipop or newer operating system or iPhone 4 or newer). The medication tray plugs into an ordinary 110 V outlet, has 28 compartments (up to 4 doses per day for 7 days), time stamps compartment use, and is capable of providing reminder signals. At the prescribed dosing day and time, a blinking blue light is activated. If, after 30 minutes, the compartment lid has not been opened and closed, a loud intermittent chime automatically activates for 30 minutes. If, at the end of those 30 minutes, the compartment lid still has not been opened, an automated reminder phone call or SMS is delivered to the subject’s mobile phone. Microelectronic circuitry in each compartment on the tray date and time stamps the opening of the compartment lid. These signals are relayed via an internal modem to the Viaca server for processing. Data will then be sent directly to a HIPAA (Health Insurance Portability and Accountability Act of 1996)-compliant relational database housed at MUSC. Personalized motivational and positive reinforcement SMS messages will be automatically delivered initially based on the previous day’s calculated medication adherence. After 2 consecutive weeks of a calculated adherence score of 1.0, message delivery will be tapered from daily to several times per week on a 3-day average variable interval schedule. The schedule will revert to daily delivery when and if a subject’s calculated weekly adherence score is <0.9. The library of motivational/social reinforcement SMS messages was developed by enhancing the library of messages used in the earlier mHealth medication adherence studies [25,26]. This step was guided by underlying tenants of competency (akin to self-efficacy) and autonomous motivation from the self-determination theory [47,48], tailored to the subject based on their responses to a questionnaire designed to identify underlying motivating themes for consistently engaging in the medical regimen.

Participants will be sent SMS messages every 3 days as a reminder to measure their BP with the Bluetooth-enabled A&D UA-767 Plus BP monitor (A&D Medical, San Jose, CA). They will be instructed to measure their BP in the morning and evening every third day by using our resting BP protocol (described under Clinic Resting Blood Pressure). They will also receive a tailored positive reinforcement SMS message the day after successful completion of their BP measurements. An app will be installed on their smartphone, which will securely receive and transmit the BP data to the MUSC-housed server. The app will also provide text instructions throughout the BP protocol and a video clip module demonstrating use of the app and the BP monitor.

**Figure 3:**
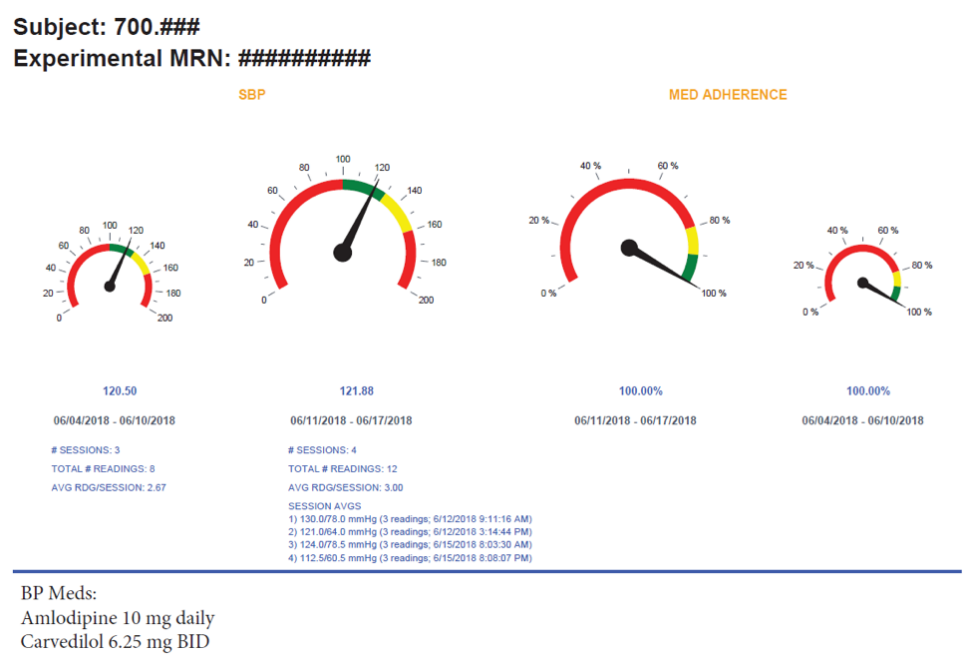
Example of a “Smartphone Medication Adherence Saves Kidneys” participant’s weekly summary report to the provider. AVG RDG: average reading, SBP: systolic blood pressure; BP: blood pressure; MRN: medical record number.

A weekly SMASK dashboard summary report, tailored to the treating physician’s preferences, will be delivered via email (Figure 3). The report will summarize each subject’s medication adherence and adherence to the BP self-monitoring schedule for the prior 2 weeks. Color coding will be used to indicate where the subject lies relative to the desired goals (ie, SBP <130 mm Hg and adherence score >0.90). In addition, a breakdown of the BP readings obtained over the 2 weeks will be provided with systolic and diastolic pressures. The treating physician will make adjustments to the medical regimen, as indicated and guided by the KDIGO guidelines [49]. The physician will notify the study coordinator of the changes via email. Any medication changes made by the treating physician will be mirrored in the programming of the medication tray after the study coordinator confirms with the patient that the changes have been enacted. The research manager and staff will be contacted via email, and the SMASK participants will be contacted via their preferred mode (SMS, email, or phone) when they fail to measure BP or when the measured BP is outside of the threshold ranges established by the treating physician, or they will be contacted via an SMS text when the SimpleMed+ tray identifies lack of medication adherence.

### Calculation of Adherence Score

A detailed description of the medication adherence score calculation by Russell et al is available elsewhere [50]. We will employ a modification of the algorithm to allow for dosing schedules other than the twice daily schedule. Our subjects will be instructed that to be considered fully adherent, their medications have to be taken within a 3-hour window of the prescribed dosing time. A dose taken within the 3-hour window will be assigned a full score for that dosing time, a dose taken outside the 3-hour window but within a 6-hour window will be assigned a half score for that dosing time, and a missed dose will be assigned a score of 0. Each subject will be assigned a score from 0.0 to 1.0 for each day. The scores for each subject will be averaged over each week.

### Identification of Medication Nonadherent Patients with Uncontrolled Hypertension

Patients who meet the initial eligibility criteria, including a resting BP evaluation (SBP ≥130 mm Hg), and provide informed consent will be enrolled in a 30-day screening period using the SimpleMed+ with its reminder functions disabled. Subjects will be given a demonstration of how to properly use the medication tray and will be required to demonstrate successful use of the tray before completion of the visit. They will receive written and oral instructions explaining that to be considered fully adherent, they must take their medications within 90 minutes of the prescribed dosing time. After confirming successful connection with the server, the tray will be programmed by the study manager to accurately reflect the subjects’ medication dosing schedule. At the conclusion of the 1-month screening period, our modification [25] of the adherence equation by Russell et al will be used to calculate a medication adherence score for each subject. Only subjects with a cumulative adherence score <0.85 across the 1-month screening period will be eligible for randomization to either the mHealth intervention group or the attention control SC group.

### Attention Control Standard Care Group

The enhanced attention control SC group will receive SC at the MUSC Kidney Transplant clinic. SC includes clinic visits as deemed appropriate, education on all matters related to posttransplantation medical care, and 24-hour phone availability of transplant coordinators. Participants randomized to the SC group will continue to use their SimpleMed+ medication tray, with its reminder functions still disabled, for an additional 6 months. To control for attention exposure, the subjects in the SC group will receive SMS messages on health-related topics excluding medication adherence. These messages include healthy lifestyle tips related to physical activity, dietary intake, nonexposure to first- or second-hand smoke, and limited alcohol intake. The SMS messages will be delivered every 3 days to approximate the schedule of the mHealth intervention group. SMS messages will include links to video or PDF content that require 3-5 minutes to review.

### Mobile Health Intervention

The participants randomized to the intervention arm will receive the SMASK mHealth system, described in detail above, for the 6-month active trial. Subjects will be provided and instructed on use of the previously validated A&D BP device, and the SMASK app will be installed on their smartphone. The escalating reminder functions of the SimpleMed+ will be enabled. The SMASK subjects will again receive written and oral instructions on adherence criteria (ie, all medications are to be taken within a 180-min window centered on the dosing time; BP is to be measured every 3 days).

SMASK subjects will complete a questionnaire on beliefs, values, and life goals with the responses used in a tree-structured algorithm to generate tailored motivational and positive reinforcement messages that are delivered to promote self-efficacy for medication adherence and autonomous regulation for sustained behavior change.

A technical support phone number will be provided in several forms (paper copy, refrigerator magnet, and SMASK app) for assistance. At the conclusion of the study, subjects will return the SimpleMed+, A&D BP monitor, and smartphone (if one was borrowed) and complete a brief questionnaire assessing their opinions of the mHealth system.

### Active Trial Evaluations and Follow-Up Phase

All subjects will be followed up for an additional 6 months following the 6-month active trial. Patients will continue to attend the clinic at a frequency determined by the provider. At 1, 3, 6, and 12 months after randomization into the SMASK or SC group, subjects will be assessed on medication adherence using medication possession ratio checks, clinic resting BPs, and completion of a brief set of questionnaires including measures to assess self-determination theory tenants of autonomous self-regulation and self-efficacy (Table 1). Providers are assessed on adherence to BP management protocols and goals. The timeliness and appropriateness of medication changes will be evaluated according to KDIGO guidelines across both groups at the conclusion of the study.

**Table 1:** List of measures and evaluation time points.

Outcome variables		Measurements/instruments used	Screening	Baseline	1 month	3 months	6 months	12 months
**Primary outcomes**								
	Medication adherence	Vaica (time-stamped)	✓	✓	✓	✓	✓	
	BP^a^	Clinic resting SBP^b^	✓	✓	✓	✓	✓	✓
**Secondary outcomes**								
	Therapeutic Inertia	Provider adherence to KDIGO^c^ goals: Timely medication changes (date of medication change following medication adherence) and BP feedback (biweekly SMASK^d^ reports for SMASK participants) and from clinic visits for all subjects (Figure 2)	Weekly^e^	Weekly^e^	Weekly^e^	Weekly^e^	Weekly^e^	Weekly^e^
	Self Determination Theory Constructs	TSRQ^f^ autonomous self-regulation [51,52] (α=0.81-0.84; correlation with GCOS^g^: *r*=0.38, *P*<.001; correlation with HCCQ^h^: *r*=0.38, *P*<.001), weight loss attendance (*r*=0.34, *P*<.001), body mass index (*r*=-0.11, *P*<.05), 18-month test-retest autonomous (*r*=0.47), controlled items (*r*_s_=0.34]) [53], correlation with medication adherence (*r*=0.58, *P*<.001)] [54]	✓			✓	✓	✓
**Exploratory outcomes**								
	eGFR^i^	GFR^j^ estimation equations [55,56]	✓	✓	✓	✓	✓	✓
	Ambulatory BP	24-h ambulatory BP with SpaceLabs 90207		✓			✓	✓
	**Methodological parameters**							
		Recruitment and retention rates: patient/provider satisfaction and usefulness scale (TSUQ^k^) [57] (satisfaction: α=0.96; usefulness: α=0.92; 1-week test-retest *r*=0.98 [58])					✓	
		Fidelity checklists: patient level (eg, connection/reloads of Vaica, BP uploads via phone, and opening of messages/education information) and provider level (eg, opening of patient summary reports and phone alerts)						
	Ancillary medication adherence measures	Medication possession rate	✓	✓	✓	✓	✓	✓
**Potential moderators/mediators**								
	Demographics	Age, education level, income, type of health care insurance	✓		✓	✓	✓	✓
	Anthropometrics	Height, weight, girth	✓	✓	✓	✓	✓	✓
	Health literacy	Health Literacy Scale [59-62], correlated with short test of functional health literacy in adults. Three questions compared to those in the Short Test of Functional Health Literacy in Adults to detect inadequate health literacy: areas under the receiver operating characteristic curve were 0.76, 0.80, and 0.87. When correlated with Short Form-36 (*r*=0.86-0.87), it differentiates between healthy status and various types of illnesses (eg, migraine vs healthy using components: physical and mental quality of life; all *P*<.001)	✓					
**Adverse events**							
	Medication Side Effects Scale [63,64]	✓	✓	✓	✓	✓	✓
	Hypertension/immunosuppression knowledge [59,65] (α=0.70, high-low correlation: *P*<.01)	✓					
	Short Form-8 (α=0.87)	✓	✓	✓	✓	✓	✓
	Perceived Stress Scale (α=0.84-0.86; 2-day test-retest *r*=0.85) [64]	✓	✓	✓	✓	✓	✓

^a^BP: blood pressure.

^b^SBP: systolic blood pressure.

^c^KDIGO: Kidney Disease Improving Global Outcomes.

^d^SMASK: Smartphone Medication Adherence Saves Kidneys.

^e^Weekly SMASK reports and from clinic visits for all subjects.

^f^TSRQ: Treatment Self-Regulation Questionnaire.

^g^GCOS: General Causality Orientations Scale.

^h^HCCQ: Health Care Communication Questionnaire.

^i^eGFR: estimated glomerular filtration rate.

^j^GFR: glomerular filtration rate.

^k^TSUQ: Telemedicine Satisfaction and Usefulness Questionnaire.

### Clinic Resting Blood Pressure

BP evaluations will be conducted at enrollment; randomization; and 1, 3, 6, and 12 months. Patients will be seated upright with right arm resting on a table at heart level and a proper cuff size will be fitted. A Dinamap Pro-Care 200 BP device (GE Healthcare, Buckinghamshire, United Kingdom) will be used to take the clinic BP measurements. The Dinamap has been validated following standard auscultatory methods from the British Hypertension Society and the Association for Advancement of Medical Instrumentation [66]. The Dinamap, as well as all other BP devices used in the trial, are calibrated according to the manufacturers’ specifications. A reading will be taken immediately, after 5 minutes of rest, and following a 2-minute interval. The average of the last two readings will be used in the analyses. Subjects in the SMASK group will use the same protocol (ie, a series of 3 BP readings with 5 and 2 minutes in between the first and second readings and between the second and third readings, respectively) at home for BP self-monitoring. This functionality will be embedded within the SMASK app and automatically guide the participants through the protocol with audio guides, timer count downs, and chimes when each waiting period is complete.

### Outcome Measures

#### Primary Outcome Measures

These include proportion of participants achieving success in meeting and sustaining adherence to the KDIGO guidelines for SBP control (resting SBP <130 mm Hg) and proportion of participants meeting and sustaining monthly medication adherence scores >0.90.

#### Secondary Outcome Measures

These include provider adherence to KDIGO guidelines, as measured by the appropriateness and timeliness of BP medication changes, and subjects’ self-report of changes in self-determination theory tenants (ie, self-efficacy and autonomous motivation).

#### Exploratory Outcome Measures

These will include changes in estimated glomerular filtration rate (eGFR) and variability of CNI trough levels (%cv). CNIs have a well-described high inter- and intrapatient variability as well as a narrow therapeutic index. For these reasons, among others, therapeutic drug monitoring of CNI is the standard of care in solid organ transplantation and guides all CNI titrations [67,68]. Changes in the 24-hour ambulatory BP will also be examined.

### Participant Timeline

A timeline for participant recruitment, intervention, and follow-up assessments is shown in Figure 1.

### Proposed Sample Size

To evaluate SMASK treatment efficacy, 80 patients will be recruited and randomly assigned to either SMASK or SC. The primary outcomes of aim 1 include the proportion of patients with >0.90 medication adherence scores (based on the date/time stamped scores from the electronic trays) and proportion of patients meeting and sustaining adherence the KDIGO guidelines for clinic-based SBP control (<130 mm Hg). In intent-to-treat (ITT) analysis, with 40 patients per group, a two-sided Chi-square test (α=0.05) will have >90% power to detect a difference of 35% in proportions of those who are medication adherent (or meeting and sustaining adherence to the KDIGO BP guidelines) in the SMASK group compared to those in the SC group at the 3-month time point. A difference of 35% in medication adherence between the groups would be considered clinically relevant and warrant changes in clinical practice. Conservative estimates for medication adherence and expected BP control proportions will be used for power calculations. This approach was taken with a small pilot study, and CIs on effect sizes were quite large. Medication adherence observed in the SMASK 3-month feasibility study was 89% (versus 0% in control group; N=19). For the clinic resting BP control, we observed that 90% versus 10% of the participants controlled BP in the SMASK and control groups, respectively. Overall attrition was 10% in the intervention group [69].

### Recruitment

Eligible patients were identified from hospital medical records by a research assistant. A research coordinator approached potential subjects in the kidney transplant clinic and obtained voluntary informed consent from patients who were interested in participating. After obtaining informed consent, the clinic resting BP protocol was conducted (see Clinic Resting Blood Pressure) and if SBP ≥130 mm Hg, the subject completed a set of questionnaires (Table 1) and was instructed to wear a SpaceLabs 90207 ambulatory BP monitor (SpaceLabs, Inc, Issaquah, WA) for 24 hours. The subject then began the 1-month screening period, as described above. The study site performs approximately 20 kidney transplants per month. We provide a smartphone and an internet data plan for the SMASK subjects who do not own a smartphone compatible with the SMASK app.

### Allocation and Concealment

Subject randomization will be stratified by race and gender and will be conducted by a statistician using a computer-generated random sequence of numbers. Participants who consent to the study and are eligible for randomization at the conclusion of the 1-month medication intake screening period are allocated to either the intervention (SMASK) or SC arm using the computer-generated randomization sequence. Each random sequence is kept concealed in an envelope that is opened by the research coordinator at the time of randomization.

### Blinding

The research assistants responsible for assessing primary and secondary outcomes will remain blinded to the patients’ group assignment throughout the study. The physician responsible for making clinical management decisions for those in the SMASK intervention arm will remain blinded to which patients are enrolled in the trial as enhanced attention SC subjects, as they are merely providing SC. However, the participants will not be blinded to their group assignment, as we provide a BP monitor and smartphone as needed to those in the intervention group.

### Data Collection and Management

Instruments for data collection are listed in Table 1. Data captured electronically will be transmitted using secure encrypted algorithms and housed on site in a secure HIPAA-compliant relational database.

### Statistical Analyses

#### Primary Outcome Measures

The ITT analysis set will be determined in accordance with the International Conference on Harmonization E9 Guideline “Statistical Principles for Clinical Trials” [70] and will include all randomized subjects. For all subjects included in the ITT analysis set, all available data points will be included in the model. We chose a mixed-effects model approach because this method is a standard method in RCTs and can handle missing data and account for correlated data such as repeated measurements within patients or patients clustered within providers [71-73].

We propose a two-level analysis strategy: Our primary analysis will include the simple mixed-effects model containing the fixed time and intervention group, time, and time-by-intervention as primary independent variables (fixed effects) and MD as a random effect to account for clustering. The main analysis will compare the two intervention groups (SMASK vs SC) at the primary time point outcome at 3 months. We will estimate medication adherence score changes and changes in resting and 24-hour BP for each subject over the trial (preintervention and 1, 3, 6, and 12 months) and the within-subject longitudinal trajectories (eg, slopes) and summarize the mean longitudinal trajectory within each group. Intraclass correlations and variance estimates will be obtained for the efficacy outcomes and covariance structure of the longitudinal scores to determine the sample size (and hence adequate power) for a future effectiveness RCT.

In secondary/exploratory analysis for aim 1 outcomes, the potential influence of *a priori* specified covariates on these models will be explored, including self-determination theory tenants, demographic and clinical characteristics, and comorbidities. In an additional exploratory analysis, effect modifications of covariates will be examined through inclusion of covariate-by-group interaction terms in the multivariable models.

#### Secondary Outcome Measures

Secondary outcome measures of changes in self-determination theory tenants (self-efficacy and autonomous self-regulation) and provider adherence to KDIGO guidelines (timing of medication changes) will be investigated using mixed effects models with these outcomes as separate dependent variables, group (SMASK vs SC) as primary independent variable, and the primary outcomes (medication adherence and clinic SBP) and clinical and demographic characteristics as adjustment variables.

#### Exploratory Outcomes Measures

In exploratory analyses, change in eGFR, variability of calcineurin trough levels, and 24-hour ambulatory BP will be compared between the two groups using pooled *t* tests (or nonparametric tests, as appropriate). If the end-of study outcomes for eGFR and 24-hour BP are missing, they will be imputed using multiple imputation methods. Further, frequency distributions of adverse events and serious adverse events will be determined, and proportions for the SMASK versus SC groups will be compared using Chi-square analyses.

### Qualitative Studies

After the conclusion of the 6-month active trial evaluation, each member of the SMASK group will be approached to participate in a key informant interview of “lived experiences” during the trial. Topic areas with probes will cover expectations, experiences, adherence, motivation, and advice from family and friends. The SMASK lead physician, the transplant nurse coordinator, and other involved staff will be invited to participate in individual interviews to assess the SMASK program from the providers’ point of view. Topics of assessment will include attitudes, barriers and facilitators for use, fidelity, and impact on therapeutic inertia. We will use the constant comparative method of qualitative analysis to code the interviews’ transcript data using NVivo 10.0. Transcripts will be independently reviewed and coded by two reviewers. Once no new themes emerge, thematic saturation will have been reached. We will compare/contrast themes from participants and providers. We will synthesize and integrate the multiple quantitative and qualitative data sources using a triangulation approach. These collective findings will guide further refinements in the SMASK system prior to our efforts to acquire external funding to enable a multisite effectiveness RCT.

## Results

The SMASK project received funding from NIDDK in June 2016, obtained institutional review board approval in April 2016, and began data collection in July 2016. As of July 2018, we have completed enrollment with a total sample size of 80 participants. Currently, we are analyzing baseline data and upon completion of analysis of the final participant in the 6-month active trial, we will begin analyzing preliminary data to be submitted for peer-review publication.

Approval for important protocol modifications will be sought from the institutional review board. Written informed consent was obtained from all patients before enrollment by a trained research coordinator. There are no anticipated risks associated with participation in the study. Patients are free to withdraw at any time. Data will be kept confidential and anonymized for analyses.

## Discussion

Despite evidence that medication nonadherence is a major contributor to suboptimal outcomes in kidney transplantation, little progress has been made toward improving medication adherence. To date, research aimed at improving medication adherence in this patient population has been hampered by the use of convenience samples, indirect measures of medication adherence, and ineffective strategies. We aim to address some of these shortcomings in this trial. mHealth technology offers an opportunity to unobtrusively monitor patients, to react in real time to indicators of patient nonadherence, and to tailor the intervention to foster likelihood of sustained adherence to the regimen. Our previous research suggests that the use of an mHealth intervention in this patient population is promising.

This study is novel in both in its design and implementation. To our knowledge, this is the first RCT involving kidney transplant recipients that aimed to evaluate the efficacy of an mHealth medical regimen self-management system (SMASK), which was developed using an iterative design process guided by synergistic tenants from behavioral and technology application theories and direct guidance from kidney transplant recipients, transplant physicians, and associated health care team members. Importantly, the SMASK program utilizes timely regimen reminder tactics with immediate feedback of the results to the patient and real-time relay of patients’ regimen engagement to a HIPPA-compliant server. This allows for the use of automated, patient-tailored, motivational, and social reinforcement messages framed upon their degree of adherence. Importantly, the health care provider is intimately involved via weekly tailored emailed subject summary reports that allow more timely medical management decisions aimed at improved medication adherence, earlier BP control, and avoidance of unnecessary escalation of care. Collectively, these strategies are directed at enhancing patients’ self-efficacy to perform the medical regimen behaviors and improving the levels of autonomous, self-directed motivation to sustain these behaviors across time with the ultimate aim of improving long-term graft survival.

There are several limitations to this study that need to be addressed. First, although the study recruits from a single transplant center, limiting its generalizability, the center is the sole transplant provider for the state of South Carolina and has a catchment population of over 4.6 million persons that encompass a wide range of ethnic, educational, and socioeconomic backgrounds. Second, while it cannot be assumed that the subjects’ willingness to use the SMASK system can be divorced entirely from the appeal of the financial compensation, we targeted the compensation at a level that would only cover the inconvenience and cost of otherwise unnecessary travel and time spent waiting. Finally, while the current study duration of only 12 months is long enough to demonstrate improvements in medication adherence and BP control, it will not be long enough to demonstrate significant improvements in graft function or graft survival. However, these outcomes will be the focus of subsequent longitudinal studies, and participants in this study will be asked to continue providing relevant data to assess longer-term outcomes.

In conclusion, this study will provide important and novel data regarding the efficacy of the mHealth SMASK system to improve medication adherence and BP control in a medication-nonadherent cohort of uncontrolled hypertensive kidney transplant recipients. An efficacious SMASK intervention would lead to a larger, multicenter, effectiveness RCT powered to evaluate clinical events in a real-world setting and with the potential to demonstrate improved outcomes at lower cost than the standard of care.

## Appendix

Multimedia Appendix 1 National Institutes of Health (NIH) peer-review reports.

## Abbreviations

CNI: calcineurin inhibitor
cv: coefficient of variation
eGFR: estimated glomerular filtration rate
ESRD: end-stage renal disease
HIPAA: Health Insurance Portability and Accountability Act
ITT: intent to treat
KDIGO: Kidney Disease Improving Global Outcomes
mHealth: mobile health
MUSC: Medical University of South Carolina
NIDDK: National Institute of Diabetes and Digestive and Kidney Diseases
SBP: systolic blood pressure
SC: standard care
SMASK: Smartphone Medication Adherence Saves Kidneys
