# Supplementary material for: “Smartphone Medication Adherence Saves Kidneys” for Kidney Transplantation Recipients: Protocol for a Randomized Controlled Trial
Source: JMIR Res Protoc. 2019 Jun 21;8(6):e13351. doi: 10.2196/13351 (PMC6611329; doi:10.2196/13351)
Supplement: Multimedia Appendix 1 [file resprot_v8i6e13351_app1.pdf]

PROGRAM CONTACT:
ANDREW NARVA
301-594-8864
narvaa@niddk.nih.gov

SUMMARY STATEMENT
(Privileged Communication)

Release Date: **06/11/2014**

Application Number: **1 R01 DK103839-01**

Principal Investigator

MCGILLICUDDY, JOHN W. MD

Applicant Organization: MEDICAL UNIVERSITY OF SOUTH CAROLINA

Review Group: **BMIO**
Behavioral Medicine, Interventions and Outcomes Study Section

Meeting Date: **06/02/2014**
Council: **OCT 2014**
Requested Start: **09/01/2014**

RFA/PA: **PA13-302**
PCC: **KAN KKT**
Dual IC(s): **HL, NR**

Project Title: **Mobile Technology Medication Adherence Program for Kidney Transplant Patients**

SRG Action: **Impact Score: 39 Percentile: 19**
Next Steps: **Visit http://grants.nih.gov/grants/next_steps.htm**
Human Subjects: 30-Human subjects involved - Certified, no SRG concerns
Animal Subjects: 10-No live vertebrate animals involved for competing appl.
Gender: **1A-Both genders, scientifically acceptable**
Minority: **1A-Minorities and non-minorities, scientifically acceptable**
Children: **3A-No children included, scientifically acceptable**
Clinical Research - not NIH-defined Phase III Trial

Project Year	Direct Costs Requested	Estimated Total Cost
1	311,992	466,428
2	299,680	448,022
3	268,866	401,955
4	287,169	429,318
TOTAL	1,167,707	1,745,723

ADMINISTRATIVE BUDGET NOTE: The budget shown is the requested budget and has not been adjusted to reflect any recommendations made by reviewers. If an award is planned, the costs will be calculated by Institute grants management staff based on the recommendations outlined below in the COMMITTEE BUDGET RECOMMENDATIONS section.

EARLY STAGE INVESTIGATOR, NEW INVESTIGATOR

1R01DK103839-01 McGillicuddy, John

EARLY STAGE INVESTIGATOR
NEW INVESTIGATOR

RESUME AND SUMMARY OF DISCUSSION: This application proposes to look at efficacy testing and refinement of an individually tailored smart phone-based medication adherence and BP monitoring program for medication non-adherence for kidney transplant recipients with uncontrolled hypertension. The area of research is very significant given the high prevalence of ESRD, the important role of transplantation, and major negative impact of non-adherence on patients' outcomes. Strengths noted include: excellent investigators and environment, the use of a RCT design, adequate preliminary data, detailed patient population and study measures, and long-term assessment of outcomes. Weaknesses are related to the management of antihypertensive medications in the intervention arm and several limitations of the proposed analysis plan and power calculation. Other weaknesses in the approach were mentioned. In summary, the committee concluded that the potential overall impact was moderate.

DESCRIPTION (provided by applicant): Despite significant advances in the care of kidney transplant recipients (KTRs), long-term graft survival remains poor. Medication non-adherence (MNA) to immunosuppressant's and uncontrolled hypertension (HTN) are predominant risk factors for premature graft rejection, graft loss, and death. Efforts to improve MNA and BP control in KTRs have met with limited success. Innovative approaches are needed that are acceptable, sustainable, efficacious, and easily disseminated. There have been no randomized controlled trials (RCTs) evaluating the application of theory-driven and individually tailored mobile health (mHealth) technology programs among KTRs with MNA and uncontrolled HTN. The proposed research will test and refine the Smart phone Medication Adherence Saves Kidneys (SMASK) program. SMASK includes multi-level components: 1) automated reminders from an electronic medication tray; 2) tailored text message/voice mail motivational feedback and reinforcement guided by self-determination theory and based upon adherence to daily medication and BP monitoring and 3) automated summary reports and direct alerts to providers. A 6-month, 2- arm (SMASK vs. enhanced Standard Care [SC]) efficacy RCT will be conducted in 116 MNA KTRs with uncontrolled HTN. Evaluations will occur at pre-intervention, months 3 and 6, and post-trial follow-ups at months 12 and 18. Specific aims are to test the hypotheses that, compared to the SC cohort, the SMASK cohort will demonstrate significantly improved and sustained changes at months 3, 6, 12, and 18 in: 1) Primary Outcome Variables: a) Medication adherence: % with electronic monitor-derived adherence scores >0.90; b) BP control: % reaching and sustaining KDIGO guidelines for BP control (clinic resting BP <130/80 mmHg). 2) Secondary Outcome Variables: a) Provider adherence to KDIGO guidelines as measured by timing of medication changes; b) Changes in Self-Determination Theory constructs (e.g., competence and autonomous regulation). 3) Exploratory Outcome Variables: a) Estimated glomerular filtration rate; b) Degree of graft fibrosis; c) Variability in calcineurin inhibitor trough levels; d) % reaching and sustaining 24-hr ambulatory BP<130/80 mmHg. After 6-month trial completion evaluation, focus groups with random sample of SMASK subjects (n=20) and healthcare providers (3-5) will assess key user reactions including acceptability, usability, salience and aids/barriers to sustainability. Data from RCT and focus groups will be triangulated to further refine and optimize SMASK and prepare for a multi-site effectiveness RCT. Our long-term objective is to reduce premature graft loss among KTRs by developing effective and sustainable mHealth secondary prevention self-management programs for medication adherence and bio-function monitoring (e.g., BP).

PUBLIC HEALTH RELEVANCE: Interventions that address medication non-adherence among kidney transplant recipients (KTRs) must be acceptable, sustainable, and easily disseminated by clinicians. We will test and further refine a smartphone delivered, tailored medical regimen self-management program, which will facilitate patient medication adherence, BP control, and clinician oversight. With

demonstrated effectiveness, the intervention will ameliorate the risk of premature graft loss among KTRs.

CRITIQUE 1:

Significance: 2
Investigator(s): 2
Innovation: 3
Approach: 5
Environment: 2

Overall Impact: This R01 Application from a new and early investigator is designed to test and refine the Smart phone Medication Adherence Saves Kidneys (SMASK) program. In US, 90,000 patients are currently listed for Transplantation while fewer than 20,000 transplants are performed annually, highlighting the need to optimize allograft longevity. It's a 6-month, 2-arm efficacy RCT that will be conducted in 116 Kidney Transplant Recipients (KTRs) with the aim of decreasing medication non-adherence to immunosuppressant's and uncontrolled HTN and overall goal to decrease the risk of premature graft rejection, graft loss and death. A pilot RCT of SMASK in 20 patients by the PI formed the foundation of the present application. The interdisciplinary investigative team has substantial experience in mhealth technologies and self-management programs and will be able to carry out the proposed project. MUSC have the necessary resources that include a top ranked Transplant Center to conduct the proposed project. Measure of impact of an adherence enhancement program on graft function markers (eGFR, and graft fibrosis), immunosuppressant medication trough level variability and 24hr ambulatory BP seems innovative. However, distracting from the enthusiasm for this application are weaknesses in some of the criteria. Randomization at subjects' level means that the 4 transplant nephrologists will have subjects in both arms of the study, a scenario that raises the possibility of contamination. The application did not demonstrate adequate direct engagement of the providers that take care of the patients in BP management and addressing the therapeutic inertia. There is no mention or engagement of caregivers in this application. Gathering exploratory information regarding the cost of the intervention would have strengthened the application. Participant burden is a minor concern and finally, the generazability of the intervention as described is a concern. The investigators will be recruiting 21-55yrs olds only thereby excluding large number of patients.

1. Significance:

Strengths

- Project aimed at efficacy testing and refinement of an individually tailored smart phone-based medication adherence and BP monitoring program for medication non-adherent for kidney transplant recipients with uncontrolled hypertension (SMASK).
- There is a need to develop more efficacious approaches to preventing the graft loss and medical co-morbidity that is attributable to medication non-adherence.
- The need to improve the extremely high rates of uncontrolled BP associated with graft dysfunction, graft loss and death among transplant patients.

Weaknesses

- None noted

2. Investigator(s):

Strengths

- The PI is a Transplant surgeon and an early and new investigator. He is currently completing his NIH KL2 Clinician Scientist award, a 3yrs clinical research training program. He developed a mHealth self –management program and administered the feasibility RCT project. His pilot RCT of 20 patients using the SMASK program formed the foundation of the present application.
- The PI has established effective collaborations with experienced investigators within the institution and his mentor is collaborating with him on this project.
- The interdisciplinary team has substantial experience in mhealth technologies and self-management programs. The team has significant experience in software/server interface development and practiced based behavioral RCTs.

Weaknesses

- None noted

3. Innovation:

Strengths

- Use of mHealth to improve transplant rejection, BP control, comorbidity and death among transplant patients
- A practitioner component specifically designed to reduce therapeutic inertia by providing the treating practitioner with information regarding subject MNA and BP levels alongside the KDIGO BP goals and MUSC stepped care guidelines for BP control in KTRs.
- Measure of impact of an adherence enhancement program on graft function markers (eGFR, and graft fibrosis), immunosuppressant medication trough level variability and 24hr ambulatory BP.

Weaknesses

- mHealth is currently been tried in different populations. Electronic monitoring and home BP measurements and feedback to provider are not entirely new.

4. Approach:

Strengths

- A 2-arm RCT design (n=116) with the subjects as the units of randomization.
- Use of Self-determination theory as the theoretical underpinning for development of SMASK.
- Use of an iterative design process guided by behavioral change theories to gather direct guidance from KTRs and health care providers
- Use of mHealth Technologies to monitor, in real time, MNA and home measured BP to facilitate delivery of immediate feedback
- Provision of healthcare providers with personalized automated summary reports to facilitate timelier medication regimens changes and earlier sustained BP control
- Inclusion of Follow –up evaluation, focus groups with SMASK providers and SMACK subjects.
- Attention control for the Standard care cohorts
- The statistical section is well developed

Weaknesses

- Randomization at subjects' level means that the 4 transplant nephrologists will have subjects in both arms of the study, a scenario that raises the possibility of contamination.
- Lack of substantial engagement of the patient's physician managing the BP in the study protocol. Only one team MD will be receiving the summary BP reports every 2 weeks and making the necessary changes per protocol. The treating physician will not be engaged actively in the process. The measurement and the usefulness of the Therapeutic inertia may not be reflective of an individual patient's provider data, since only one MD is receiving all the summary BP reports and making changes to the medication.
- There are so many messages and reminders, (multiple reminders to check BP, escalating reminders to take medications using the MedMinder (from light to chime to cell phone calls/SMS), this may lead to participant fatigue or overburden. The patient should be asked how often they will like to get a reminder as part of the design.
- Messages are going to be customized to each individual patient based on their belief, values and goals this reviewer questions the generazability of this process beyond the intervention period.
- There is no mention or engagement of caregivers in this application. One of the inclusion criteria is that the patient should be able to take their own BP. What if the patient can't but has a caregiver that can help to do that?
- Lack of exploratory aim to information regarding the cost of the intervention

5. Environment:

Strengths

- The MUSC have the necessary resources that include a top ranked Transplant Center to conduct the proposed project.
- The SC Clinical and Translational Research Institute, Office of Biomedical Informatics Systems and The Technology Applications Center for Healthful Lifestyles (TACHL) are available to support this project.

Weaknesses

- None noted

Protections for Human Subjects:

Acceptable Risks and/or Adequate Protections

Data and Safety Monitoring Plan (Applicable for Clinical Trials Only):

Acceptable

Inclusion of Women, Minorities and Children:

G1A - Both Genders, Acceptable

M1A - Minority and Non-minority, Acceptable

C3A - No Children Included, Acceptable

- 50% of the participants will be women and minority will be included, 60% of the participants will be African American

Vertebrate Animals:

Not Applicable (No Vertebrate Animals)

Biohazards:

Not Applicable (No Biohazards)

Budget and Period of Support:

Recommend as Requested

CRITIQUE 2:

Significance: 3
Investigator(s): 2
Innovation: 2
Approach: 4
Environment: 1

Overall Impact: The application will evaluate an integrated intervention to improve medication adherence and blood pressure control in post kidney transplant patients. The area of research is very significant given the high prevalence of ESRD, the important role of transplantation, and major negative impact of non-adherence on patients' outcomes. The PI is a new investigator with adequate training, he is well prepared to lead the study, the team is multidisciplinary and resources are excellent. The study is innovative in testing an integrated monitoring system with reporting to providers to improve medication adherence and blood pressure control. Strengths of the proposal include the use of a randomized controlled design, adequate preliminary data, detailed patient population and study measures, and long-term assessment of outcomes. Weaknesses are related to the management of antihypertensive medications in the intervention arm and several limitations of the proposed analysis plan and power calculation. Overall, strengths outweigh study weaknesses.

1. Significance:

Strengths

- ESRD is a significant public health problem and transplantation is an important form of treatment
- Medication non-adherence is a major cause of transplant rejection (30%-40% of all cases), graft loss, uncontrolled hypertension and death among renal transplant recipients
- Current approaches to non-adherence are only partially successful leaving many transplant recipients at risk for poor outcomes

Weaknesses

- Some weaknesses in the design and statistical analysis may limit the validity and impact of study findings

2. Investigator(s):

Strengths

- The PI is a new investigator with excellent clinical training. He recently completed a KL-2 award and lists 15 relevant publications (4 as first author; total number of publications is not provided)
- The team of investigators is multidisciplinary with experts in medicine, health communications, transplant surgery, nursing, pharmacology, biostatistics, and information technology
- There are prior collaborations among researchers

Weaknesses

- None noted

3. Innovation:

Strengths

- Will use a multi-component, integrated program to monitor in real time medication adherence and BP control providing these data to the treating team
- Use of personalized messages is somewhat novel

Weaknesses

- Some components have been previously used to improve medication adherence in other chronic conditions

4. Approach:

Strengths

- Good preliminary data regarding the effectiveness of the intervention from pilot study of 20 hypertensive kidney transplant recipients
- The intervention was developed using the theory of using self-determination. The theoretical framework is well integrated into the measurement model and data analysis plan
- Will use an RCT design with intention to treat analysis ensuring strong internal validity of study findings. Balanced attention control
- Adequate inclusion/exclusion criteria
- Will assess long-term outcomes (up to 18 months post randomization)
- Well described outcome measures. Will use objective measures of medication adherence and laboratory/pathological measures of renal function and graft fibrosis

Weaknesses

- A single transplant physician will receive all adherence reports and prescribe antihypertensive medications. This investigator may be highly invested in the success of the trial and not blinded to study arm (as reports will be only generated for those randomized to intervention) potentially introducing bias. One of secondary outcomes is therapeutic inertia (operationalized as adherence to KDIGO guidelines), however; there will be a substantial unbalance in the way BP is managed in both arms (single investigator vs. usual care)
- Unclear if other drugs (besides hypertension medications and immunosuppressive drugs) will be managed with the MedMinder
- Rationale for 24 hour ambulatory blood pressure monitoring is not provided. Unclear if daily home blood pressure monitoring will be limited to the intervention arm

- Limited information is provided on study measures and instruments
- Sample size calculations do not consider dropouts, the study will be only powered to detect large differences in adherence (OR: 4)
- Data analysis proposes to include a random physician effect but patients in the intervention arm will be primarily managed by a single provider. Plans to include mediators as covariates in adjusted analysis of treatment effect are problematic. Aim 2: inclusion of outcomes (BP and adherence) as predictors in the model assessing SDT constructs would lead to endogeneity

5. Environment:

Strengths

- Resources at the University of South Carolina are excellent

Weaknesses

- None noted

Protections for Human Subjects:

Acceptable Risks and/or Adequate Protections

Data and Safety Monitoring Plan (Applicable for Clinical Trials Only): appropriate

Inclusion of Women, Minorities and Children:

G1A - Both Genders, Acceptable

M1A - Minority and Non-minority, Acceptable

C3A - No Children Included, Acceptable

- While a large percentage of the population will be Black, there will be no representation of Hispanics. No justification is provided

Vertebrate Animals:

Not Applicable (No Vertebrate Animals)

Biohazards:

Not Applicable (No Biohazards)

Budget and Period of Support:

Recommended as Requested

CRITIQUE 3:

Significance: 2
Investigator(s): 3
Innovation: 4
Approach: 3

Environment: 3

Overall Impact: Failure to adhere to medication regimen and uncontrolled hypertension are significant risk factors for mortality and morbidity among kidney transplant recipients. Interventions to date have had limited success, and using self-determination theory (SDT) as a conceptual framework, the authors propose a system using smart phone reminders, summary reports, and alerts sent directly to providers to address the problem of long-term graft survival. This may be associated with cognitive impairment, including forgetfulness and poor planning. Given that the medical regimen is typically complex (12-15 medications up to four times a day, with adherence to timing an important variable). One estimate is that about half of transplant recipients have smart phones, and the investigators plan to use these devices for real-time interaction with non-adherent patients. Although the use of mHealth technology for the management of a chronic condition is not novel, its use with kidney transplant recipients (KTRs) has not previously been evaluated. Preliminary research by the study team found a high prevalence of medication errors by patients, and poorly controlled hypertension, and a feasibility trial of their program showed improvements in both systolic blood pressure and adherence in the intervention group. They propose a randomized controlled trial (RCT) with a total sample of 116 persons. The smart phone and an electronic medication tray (MedMinder) will be used to monitor medications and provide the patient with reminder about medication and blood pressure measurement. Outcomes will be measured at 3, 6, 12, and 18 months. Overall, the clinical trial is well-designed with meaningful long-term follow-up, but it has some limitations. The likelihood of missing data and attrition is relatively high, especially given the prevalence of cognitive impairment (especially of executive functioning) in the sample. Given that estimates of cognitive impairment among persons with ESRD range from about 16% to nearly 40%, and that executive function deficits may adversely affect subjects' ability to regulate their own behavior. Hence, a substantial portion of the sample may have difficulty following through with the intervention. In addition, the investigators may be missing an important mediating variable in their analyses. Overall impact of the study is likely to be good.

Protections for Human Subjects:

Acceptable Risks and/or Adequate Protections

- Minimal risk with exception of renal biopsies, which are standard of clinical care.

Data and Safety Monitoring Plan (Applicable for Clinical Trials Only):

Not Applicable (No Clinical Trials)

Inclusion of Women, Minorities and Children:

G1A - Both Genders, Acceptable

M1A - Minority and Non-minority, Acceptable

C3A - No Children Included, Acceptable

- 58% female, 60% Black 0% Hispanic/Latino, no children

Vertebrate Animals:

Not Applicable (No Vertebrate Animals)

Biohazards:

Not Applicable (No Biohazards)

Budget and Period of Support:

Recommend as Requested

THE FOLLOWING RESUME SECTIONS WERE PREPARED BY THE SCIENTIFIC REVIEW OFFICER TO SUMMARIZE THE OUTCOME OF DISCUSSIONS OF THE REVIEW COMMITTEE ON THE FOLLOWING ISSUES:

PROTECTION OF HUMAN SUBJECTS (Resume): ACCEPTABLE
No concerns.

INCLUSION OF WOMEN PLAN (Resume): ACCEPTABLE
58% female

INCLUSION OF MINORITIES PLAN (Resume): ACCEPTABLE
While a large percentage of the population will be Black (60%), there will be no representation of Hispanics. No justification is provided

INCLUSION OF CHILDREN PLAN (Resume): ACCEPTABLE
Children are excluded since there is a low incidence of kidney transplant in that age group and issues for children are different from adults.

COMMITTEE BUDGET RECOMMENDATIONS: The budget was recommended as requested.

SCIENTIFIC REVIEW OFFICER'S NOTES:
The NIH special practice for new investigator R01 applications reviewed in the Center for Scientific Review study sections applies to this application. Resubmission (amended -A1) R01 applications from new investigators may be submitted on a special receipt date for review in the very next review cycle. See this notice in the NIH Guide for Grants and Contracts for more details: http://grants1.nih.gov/grants/guide/notice-files/NOT-OD-11-057.html.

You should contact the NIH program officer whose name is shown in the upper left hand corner of page one of this Summary Statement for information about whether this application may be fundable or whether you will need to submit an amended application. The program officer can also help you decide whether the changes and improvements necessary to address the weaknesses noted in the reviewers' critiques could be accomplished in the relatively short time available. You are also strongly advised to seek input from mentors, your Department chair, etc.

If you choose to submit a resubmission application for the next review cycle under this policy for new investigators, your amended application must be received at NIH no later than Monday, August 11, 2014.

You may, of course, choose to take more time to resubmit your application. If so, you should prepare the resubmission for the normal dates for amended applications as specified in this table: http://grants1.nih.gov/grants/funding/submissionschedule.htm.

NIH has modified its policy regarding the receipt of resubmissions (amended applications). See Guide Notice NOT-OD-10-080 at http://grants.nih.gov/grants/guide/notice-files/NOT-OD-10-080.html.

The impact/priority score is calculated after discussion of an application by averaging the overall scores (1-9) given by all voting reviewers on the committee and multiplying by 10. The criterion scores are submitted prior to the meeting by the individual reviewers assigned to an application, and are not discussed specifically at the review meeting or calculated into the overall impact score. Some applications also receive a percentile ranking. For details on the review process, see http://grants.nih.gov/grants/peer_review_process.htm#scoring.

MEETING ROSTER

Behavioral Medicine, Interventions and Outcomes Study Section
Risk, Prevention and Health Behavior Integrated Review Group
CENTER FOR SCIENTIFIC REVIEW
BMIO
June 02, 2014 - June 03, 2014

CHAIRPERSON

FRANCE, CHRISTOPHER R, PHD
PROFESSOR
DEPARTMENT OF PSYCHOLOGY
OHIO SATE UNIVERSITY
ATHENS, OH 45701

MEMBERS

AIKENS, JAMES E, PHD *
ASSOCIATE PROFESSOR
DEPARTMENT OF FAMILY MEDICINE
UNIVERSITY OF MICHIGAN
ANN ARBOR, MI 48109

DIEFENBACH, MICHAEL A, PHD *
ASSOCIATE PROFESSOR
DEPARTMENT OF UROLOGY AND ONCOLOGICAL SCIENCES
ICAHN SCHOOL OF MEDICINE AT MOUNT SINAI
NEW YORK, NY 10029

ESTABROOKS, PAUL , PHD
PROFESSOR
DEPARTMENTS OF HUMAN NUTRITION, FOODS AND EXERCISE
CENTER FOR TRANSLATIONAL OBESITY RESEARCH
VIRGINIA POLYTECHNIC INSTITUTE & STATE UNIVERSITY
ROANOKE, VA 24016

FITZGIBBON, MARIAN L, PHD
PROFESSOR
DEPARTMENT OF MEDICINE
UNIVERSITY OF ILLINOIS AT CHICAGO
CHICAGO, IL 60608

GRIGSBY, JAMES P, PHD
PROFESSOR
DEPARTMENTS OF MEDICINE
AND PSYCHOLOGY
UNIVERSITY OF COLORADO
DENVER, CO 80217

HAMILTON, ROY H, MD
ASSISTANT PROFESSOR
DEPARTMENT OF NEUROLOGY AND
PHYSICAL MEDICINE AND REHABILITATION
HOSPITAL OF THE
UNIVERSITY OF PENNSYLVANIA
PHILADELPHIA, PA 19104

HOULE, TIMOTHY T, PHD
ASSOCIATE PROFESSOR
DEPARTMENT OF ANESTHESIOLOGY
WAKE FOREST UNIVERSITY
WINSTON-SALEM, NC 27157

KARP, JORDAN F, MD
ASSOCIATE PROFESSOR
DEPARTMENT OF PSYCHIATRY
UNIVERSITY OF PITTSBURGH
PITTSBURGH, PA 15213

KRULL, KEVIN R, PHD *
MEMBER
DEPARTMENT OF EPIDEMIOLOGY
AND CANCER CONTROL
ST. JUDE CHILDREN'S RESEARCH HOSPITAL
MEMPHIS, TN 38105

LENGACHER, CECILE A, PHD
PROFESSOR AND DIRECTOR
COLLEGE OF NURSING
UNIVERSITY OF SOUTH FLORIDA
TAMPA, FL 336124766

MILLER, SUZANNE M, PHD
DIRECTOR
DEPARTMENT OF PSYCHOSOCIAL
AND BEHAVIORAL MEDICINE
FOX CHASE CANCER CENTER
AND TEMPLE HEALTH
PHILADELPHIA, PA 19111

NAYLOR, MAGDALENA R, MD, PHD
PROFESSOR
DEPARTMENT OF PSYCHIATRY
UNIVERSITY OF VERMONT
BURLINGTON, VT 05401

OLOMU, ADE B, MD, MS
PROFESSOR
DEPARTMENT OF MEDICINE
MICHIGAN STATE UNIVERSITY
EAST LANSING, MI 48824

PALERMO, TONYA M, PHD
PROFESSOR
SEATTLE CHILDREN'S HOSPITAL
RESEARCH INSTITUTE
SEATTLE, WA 98145

RAVENELL, JOSEPH E, MD *
ASSISTANT PROFESSOR
DIVISION OF GENERAL INTERNAL MEDICINE
NEW YORK UNIVERSITY SCHOOL OF MEDICINE
NEW YORK , NY 10016

RIEKERT, KRISTIN A, PHD
ASSOCIATE PROFESSOR
DIVISION OF PULMONARY
AND CRITICAL CARE MEDICINE
JOHNS HOPKINS SCHOOL OF MEDICINE
BALTIMORE, MD 21224

RITZ, THOMAS E, PHD
PROFESSOR
DEPARTMENT OF PSYCHOLOGY
SOUTHERN METHODIST UNIVERSITY
DALLAS, TX 75275

RUIZ, JOHN M, PHD
ASSOCIATE PROFESSOR
DEPARTMENT OF PSYCHOLOGY
CLINICAL HEALTH PSYCHOLOGY
UNIVERSITY OF NORTH TEXAS
DENTON, TX 76203

WISNIVESKY, JUAN P, MD, DPH
PROFESSOR
DEPARTMENT OF MEDICINE
ICAHN SCHOOL OF MEDICINE AT MOUNT SINAI
NEW YORK, NY 100296574

WYSOCKI, TIM , PHD
CO-DIRECTOR
CENTER FOR HEALTH CARE DELIVERY SCIENCE
NEMOURS CHILDREN'S HEALTH SYSTEM
JACKSONVILLE, FL 32207

SCIENTIFIC REVIEW ADMINISTRATOR

MANN, LEE S, PHD
SCIENTIFIC REVIEW OFFICER
CENTER FOR SCIENTIFIC REVIEW
NATIONAL INSTITUTES OF HEALTH
BETHESDA, MD 20892

GRANTS TECHNICAL ASSISTANT

WATTS, MELISSA D
EXTRAMURAL SUPPORT ASSISTANT
CENTER FOR SCIENTIFIC REVIEW
NATIONAL INSTITUTE FOR HEALTH
BETHESDA, MD 20892

* Temporary Member. For grant applications, temporary members may participate in the entire meeting or may review only selected applications as needed.

Consultants are required to absent themselves from the room during the review of any application if their presence would constitute or appear to constitute a conflict of interest.
